# Projecting social contact matrices to different demographic structures

**DOI:** 10.1371/journal.pcbi.1006638

**Published:** 2018-12-07

**Authors:** Sergio Arregui, Alberto Aleta, Joaquín Sanz, Yamir Moreno

**Affiliations:** 1 Institute for Biocomputation and Physics of Complex Systems (BIFI), University of Zargoza, Zargoza, Spain; 2 Department of Theoretical Physics, University of Zaragoza, Zaragoza, Spain; 3 Department of Genetics. Saint-Justine Hospital Research Center, Montreal, Canada; 4 Department of Biochemistry, University of Montreal, Montreal, Canada; 5 ISI Foundation, Turin, Italy; Max-Planck-Institute for Evolutionary Biology, GERMANY

## Abstract

The modeling of large-scale communicable epidemics has greatly benefited in the last years from the increasing availability of highly detailed data. Particullarly, in order to achieve quantitative descriptions of the evolution of epidemics, contact networks and mixing patterns are key. These heterogeneous patterns depend on several factors such as location, socioeconomic conditions, time, and age. This last factor has been shown to encapsulate a large fraction of the observed inter-individual variation in contact patterns, an observation validated by different measurements of age-dependent contact matrices. Recently, several works have studied how to project those matrices to areas where empirical data are not available. However, the dependence of contact matrices on demographic structures and their time evolution has been largely neglected. In this work, we tackle the problem of how to transform an empirical contact matrix that has been obtained for a given demographic structure into a different contact matrix that is compatible with a different demography. The methodology discussed here allows to extrapolate a contact structure measured in a particular area to any other whose demographic structure is known, as well as to obtain the time evolution of contact matrices as a function of the demographic dynamics of the populations they refer to. To quantify the effect of considering time-dynamics of contact patterns on disease modeling, we implemented a Susceptible-Exposed-Infected-Recovered (SEIR) model on 16 different countries and regions and evaluated the impact of neglecting the temporal evolution of mixing patterns. Our results show that simulated disease incidence rates, both at the aggregated and age-specific levels, are significantly dependent on contact structures variation driven by demographic evolution. The present work opens the path to eliminate technical biases from model-based impact evaluations of future epidemic threats and warns against the use of contact matrices to model diseases without correcting for demographic evolution or geographic variations.

## Author summary

Large scale epidemic outbreaks represent an ever increasing threat to humankind. In order to anticipate eventual pandemics, mathematical modeling should not only have the capacity to model in real time an ongoing disease, but also to predict the evolution of potential outbreaks in different locations and times. To this end, computational frameworks need to incorporate, among other ingredients, realistic contact patterns into the models. This not only implies anticipating the demographic structure of the populations under study, but also understanding how demographic evolution reshapes social mixing patterns along time. Here we present a mathematical framework to solve this problem and test our modeling approach on 16 different empirical contact matrices. We also evaluate the impact of an eventual future outbreak by simulating a SEIR scenario in the countries and regions analyzed. Our results show that using outdated or imported contact matrices that do not take into account demographic structure or its evolution can lead to largely misleading conclusions.

## Introduction

During recent years, models on disease transmission have improved in complexity and depth, integrating high-resolution data on demography, mobility and social behavior [1, 2]. Specifically, the topology of social contacts plays a major role in state-of-the-art modeling [3–8]. The complete knowledge of the network of contacts through which an epidemic spreads is usually unreachable or impossible to implement, and for modeling purposes it is useful to remain at the coarse level of age-groups. Under this view, the population under study is divided into different groups according to its age distribution and different contact rates are assumed among these groups. Age-dependent contact patterns give powerful insights on the transmission of diseases where epidemiological risk is age-dependent, either as a result of behavioral or physiological factors. Relevant examples are influenza-like diseases [6–10], pertussis [11], tuberculosis [12, 13], and varicella [14]. Furthermore, they are instrumental for modeling and implementing more efficient interventions [15, 16].

Given the utmost importance of contact heterogeneities, the study of age-dependent social mixing has become a priority in the field. In 2008, Mossong et al. [17] published a seminal work with the measurements of age-dependent contact rates in eight European countries (Belgium, Finland, Germany, Great Britain, Italy, Luxembourg, Netherlands and Poland) via contact diaries. Due to the high cost of gathering empirical data on social contacts, Fumanelli et al. [18] proposed an alternative path consisting on building synthetic contact patterns via the modelling of virtual populations. Nevertheless, other authors have followed the original route opened by Mossong et al., measuring empirically the age-dependent social contacts of other countries such as China [19], France [20], Japan [21], Kenya [22], Russia [23], Uganda [24] or Zimbabwe [25], as well as the Special Administrative Region of Hong Kong [26], thus expanding significantly the available data on social mixing in the last few years. In these studies, participants are asked how many contacts they have during a day and with whom. This allows to obtain the (average) number of contacts that an individual of a particular age *i* has with individuals of age-group *j*. The resulting matrix is not symmetric due to the different number of individuals in each age-group. However, it is precisely the demographic structure that imposes constraints in the entries of this matrix, as reciprocity of contacts should be fulfilled at any time (i.e., the total number of contacts reported by age-group *i* with age-group *j* should be ideally equal in the opposite direction). Therefore, an empirical contact matrix, that has been measured on a specific population, should not be used directly, without further considerations, in another population with a different demographic structure.

This issue has important consequences in the field of disease modeling. As contact matrices play a key role in disease forecast, it is essential to assure that the matrices implemented are adapted to the demographic structure of the population considered in order to avoid biased estimations. For some short-cycle diseases like influenza, the time scale of the epidemic is much shorter than the typical times needed for a demographic structure to evolve. That means that, typically, the demographic structure can be safely considered constant [10], and the eventual evolution of the contact matrix can be neglected throughout the simulation of an outbreak. For these diseases, the problems might arise when modelers use contact matrices that are not up to date -for instance, one might wonder whether the patterns reported in [17] in 2008 can be used nowadays, a decade later, during which all the European countries analyzed in that study aged significantly. The same issue appears when a contact matrix measured in a given location (e.g., a specific country) is directly used to simulate disease spreading in another region or country with a different population structure.

The previous considerations are even more troublesome for the case of persistent diseases that need long-term simulations, for which the hypothesis of constant demographic structures does not hold anymore [12]. In those cases, contact matrices should continuously evolve during the simulation to reflect the effect that an evolving demography should exert on contact structures. Furthermore, it remains unknown to what extent the variations between contact matrices coming from different geographic settings are due to differences in the demographic structures, divergent cultural traits and/or methodological differences between studies. For instance, elderly people exhibit higher contact rates with children in African countries than in Europe [25]. This could be explained by the different demographic structures: one might expect to observe higher contact rates toward the younger age strata in Africa than in Europe because their populations have a higher density of young individuals. However, it is not clear yet whether the demographic structure is the only driver of geographical heterogeneity between empirical contact matrices.

The problems that arise when exporting contact patterns across settings have been noticed in previous studies, specially in what concerns matrix reciprocity. Recently, in [27], Prem et al. proposed a method to export European contact patterns to different settings around the world in a way that preserves reciprocity. Similarly, in other epidemiological studies, when implementing heterogenous contact patterns, modelers apply different corrections to solve the problem of non-reciprocity [7, 8, 11, 28, 29]. However, a general discussion on the side implications of these corrections and their range of applicability is still missing.

The main focus of this work is to study how age contact matrices, originally obtained for a specific setting (country and year), can be adapted to different demographic structures -i.e., to another (location and/or time) setting. To this end, we first study the magnitude of the reciprocity error incurred when the adaptation of empirical social contacts to different age structures is ignored, thus justifying the need of studying possible projections that solve this problem. Next, we analyze different methods to perform these adaptations, highlighting the differences induced in the contact patterns by the use of these methods. We also compare empirical contact matrices of 16 countries and regions in different areas worldwide filtering the influence of the demographic structure. This allows us to isolate the differences between contact patterns that are caused by any other factors, such as socio-cultural traits or methodological aspects, from those caused by demographic variability across settings. Finally, we implement a Susceptible-Exposed-Infected-Recovered (SEIR) dynamics to study the differences in prospected incidences that arise when applying the methods analyzed to project social contact matrices.

## Materials and methods

### Collection of empirical survey matrices

For this work we have gathered 16 different contact matrices coming from several geographic settings: 8 from the POLYMOD project [17] (Belgium, Finland, Germany, Great Britain, Italy, Luxembourg, Netherlands and Poland), China [19], France [20], Hong-Kong [26], Japan [21], Kenya [22], Russia [23], Uganda [24] and Zimbabwe [25].

There are some methodological differences between these studies, thus some pre-processing to homogenize the matrices is required. Specifically, we need to transform them to the same definition of contact matrix and adapt them to the same age-groups. Once this is done, we perform a reciprocity correction (valid for the demographic structure corresponding to the country and year where the survey took place), and we normalize the matrices so that the mean connectivity is equal to one. Details can be found in the Supplementary Information.

### Demographic data

Data regarding the time evolution of demographic structures, either observed in the past or projected until 2050, have been retrieved from the UN population division database [30].

### Projections of a contact matrix

The basic problem explored in this work is: how can we transform the (empirical) contact matrix *M*_*i*,*j*_, that has been measured for a specific demographic structure *N*_*i*_, into a different contact matrix $M_{i,j}^{\prime }$ that is compatible with a different demographic structure $N_{i}^{\prime }?$ This could mean to adapt data obtained in one specific country to another different region that has a different demography. But the problem can appear even if we remain in the same geographical setting, as a contact matrix measured at a specific time *τ*, could not be valid for an arbitrary time *t* if the demographic structure of that population has changed. In the following sections, we formulate the problem of non-reciprocity and we present and discuss different methods of using contact matrices in an arbitrary demographic structure.

#### Method 0 (M0): Unadapted contact matrix. The problem of non-reciprocity

We will call *M*_*i*,*j*_ to the mean number of contacts that an individual of age *i* has with other individuals of age *j* during a certain period of time. This is the magnitude that is usually reported when contact patterns are measured empirically [17, 19–23, 26]. The number of contacts must fulfil reciprocity, i.e., there is the same number of total contacts from age-group *i* to age-group *j* than from *j* to *i*. This imposes the following closure relation for the contact matrix:

$$\begin{array}{c} M_{i,j}N_{i}=M_{j,i}N_{j}\Rightarrow \frac{M_{i,j}}{M_{j,i}}=\frac{N_{j}}{N_{i}} \end{array}$$ (1)

where *N*_*i*_ is the number of individuals of age-group *i*.

Therefore, in the case of an evolving demographic structure for which the ratio $\frac{N_{i}}{N_{j}}$ is not constant; the contact matrix *M*_*i*,*j*_ must change with time. Otherwise we will have non-reciprocal contacts (contacts that inconsistently appear in one direction but not in the other). When comparing different methods for correcting for reciprocity we will usually also compare with the case in which this problem is completely ignored, and the matrix *M*_*i*,*j*_ is taken directly from the survey without any further consideration. We will refer to this case as Method 0 (M0).

The following methods correct this problem, introducing different transformations of the original contact matrix *M*_*i*,*j*_, that was measured in a demographic structure *N*_*i*_, into a new contact matrix $M_{i,j}^{\prime }$ that is well adapted to a new demographic structure $N_{i}^{\prime }$ (at least avoiding the problem of no reciprocity).

#### Method 1 (M1): Pair-wise correction

The basic problem that we want to avoid is to have a different number of contacts measured from *i* to *j* than from *j* to *i*. Thus, an immediate correction would be to simply average those numbers, so the excess of contacts measured in one direction is transferred to the reciprocal direction. This correction can be formulated as:

$$\begin{array}{c} M_{i,j}^{\prime }=\frac{1}{N_{i}^{\prime }}\frac{1}{2}(M_{i,j}N_{i}^{\prime }+M_{j,i}N_{j}^{\prime })=M_{i,j}\frac{1}{2}(1+\frac{N_{i}N_{j}^{\prime }}{N_{j}N_{i}^{\prime }}) \end{array}$$ (2)

#### Method 2 (M2): Density correction

An alternative approach is to adapt contact patterns to different demographic structures correcting by the density of available contactees, which we formalize with the following equation:

$$\begin{array}{c} M_{i,j}^{\prime }=\Gamma_{i,j}\frac{N_{j}^{\prime }}{N^{\prime }} \end{array}$$ (3)

Thus, we interpret that the matrix *M*_*i*,*j*_ is the product of two factors:

- The intrinsic connectivity matrix: Γ_*i*,*j*_
- The fraction of individuals in *j*: $\frac{N_{j}^{\prime }}{N^{\prime }}$

Thus, we are assuming that an individual has an intrinsic preference over certain age-groups depending on its age, captured by Γ_*i*,*j*_ and the final contact rate is modified according to the density of available contactees.

The matrix Γ_*i*,*j*_ corresponds, except for a global factor, to the contact pattern in a “rectangular” demography (a population structure where all age groups have the same density). We can obtain these matrices Γ_*i*,*j*_, that are country-specific, from survey data using Eq 3:

$$\begin{array}{c} \Gamma_{i,j}=M_{i,j}\frac{N}{N_{j}} \end{array}$$ (4)

which allows to rewrite Eq 3 as a function of the original matrix *M*_*i*,*j*_:

$$\begin{array}{c} M_{i,j}^{\prime }=M_{i,j}\frac{NN_{j}^{\prime }}{N_{j}N^{\prime }} \end{array}$$ (5)

This methodology for adapting contact patterns has already been used by De Luca and collaborators, introducing the matrix Γ_*i*,*j*_ in the force of infection [8]. Also a similar correction is used in Prem et al. [27] to adapt European contact matrices to other countries (although this work integrates more data beyond demographic structures).

#### Method 3 (M3): Density correction + normalization

A cardinal feature of M2 is that it does not preserve the mean connectivity of the entire network of contacts. As a result, depending on the initial contact matrix and the dynamics of the demography, the evolution of the contact structure can produce average connectivities that depart strongly from its initial value. Although considering an evolution of the mean connectivity as demography changes might be reasonable, the inability of M2 of producing contact matrices of stable mean connectivities might be considered a liability in some scenarios.

Taking that potential issue into consideration, we have proposed an alternative approach that, in addition of correcting for the densities of contactees, preserves the mean connectivity of the overall system across time. Thus, an evolution of the mean connectivity could always be forced by adding a global factor in a controlled way.

To do so, we begin by defining ${\tilde{M}}_{i,j}$ as the connectivity matrix from M2:

$$\begin{array}{c} {\tilde{M}}_{i,j}=\Gamma_{i,j}\frac{N_{j}^{\prime }}{N^{\prime }} \end{array}$$ (6)

and then we divide it by its connectivity:

$$\begin{array}{c} M_{i,j}^{\prime }=\frac{{\tilde{M}}_{i,j}}{<\tilde{k}>} \end{array}$$ (7)

Thus:

$$\begin{array}{c} M_{i,j}^{\prime }=\frac{\Gamma_{i,j}N_{j}^{\prime }N^{\prime }}{\sum_{i,j}\Gamma_{i,j}N_{i}^{\prime }N_{j}^{\prime }}=M_{i,j}\frac{N_{j}^{\prime }}{N_{j}}\frac{N^{\prime }}{\sum_{i,j}M_{i,j}\frac{N_{i}^{\prime }N_{j}^{\prime }}{N_{j}}} \end{array}$$ (8)

Notice that all methods trivially coincide in the year in which the data was obtained (i.e. when the survey was done). Also the definition of Γ_*i*,*j*_ does not change between M2 and M3 in these cases, as the initial *M*_*i*,*j*_ has been normalized to have a mean degree of 1, and we extract it with the same equation as before (Eq 4).

### Overview of different methods

Summing up, in this work we discuss up to four different methods in order to adapt contact patterns estimated in a given setting to a different one for which there is no available data. In Table 1 we provide a summary of the main properties of each method.

**Table 1:** Properties of different methods.

Method	Reciprocity?	Preserves Intrinsic Connectivity?	Constant average connectivity?
M0: Unadapted contact patterns	No	No	No
M1: Pair-wise correction	Yes	No	No
M2: Density correction	Yes	Yes	No
M3: Density correction + Normalization	Yes	Yes (with a global factor)	Yes

Summary of the different methods to deal with contact patterns and their properties.

The first of them, called M0, consists of applying the original contact structures available on the setting to study with no correction. This, as previously discussed, leads to contact structures that violate the requirement of total contacts reciprocity. A second approach, called M1, consists of a direct correction of the reciprocity bias, which suffers however from another conceptual issue, namely, it does not preserve intrinsic connectivity. This means that, under M1, the number of contacts that an individual in age-group *i* has per unit time with individuals in another age-group *j*, will not be proportional to the density of available contactees in *j* when adapting the matrix across settings. Considering these conceptual limitations, these two elementary approaches should be avoided whenever demographic data is available, in favour of alternative methods such as M2 or M3.

As for M2 and M3, the main difference between them involves the presence or absence of a global factor multiplying the entire contact matrix when comparing their outcomes on the same setting. While both methods similarly respect reciprocity and intrinsic connectivity requirements, overall connectivity is not preserved under M2, but it is under M3. Concerning their application to disease transmission modelling, the relevance of this difference depends on the modelling context.

On the one hand, we have situations where an incipient epidemic phenomenon starts in a setting that is different -either in time or space- from the one where contact data is available, and its basic infectiousness has to be calibrated from its early stages using a transmission model. This usually happens with emergent diseases, yet uncharacterised, as well as with pathogens whose transmission dynamics is highly variable due to high mutation rates (typically virus). In these contexts, modelers are forced to re-calibrate global infectiousness, among other key epidemiological parameters, for every outbreak. Also, if the typical duration of the outbreak is smaller than the time-scale during which demographic dynamics occurs (e.g. from weeks to months), then contact structures can be safely considered invariant during the simulation of the event. In these contexts, using M2 or M3 leads to largely similar outbreak descriptions. The reason is that the independent calibration of the infectiousness at the beginning of the outbreak absorbs the changes in global connectivity that are the only difference between the contact matrices produced by M2 or M3. This means that, under this scenario, the main difference between the methods will translate into the inference of arbitrarily different infectiousness parameters after model calibration to describe the same epidemic event. A paradigmatic example of this kind of situation is the modeling of seasonal influenza, that typically involves calibration of each year strains’ infectiousness at the early onset of the season outbreak.

In other contexts, whenever real-time model calibration is not an option, or the epidemic simulations need to extend over time periods that are not short enough to exclude demographic dynamics (e.g. from years to decades), the lack of control that M2 provides regarding overall connectivity makes more advisable the usage of M3. One cardinal example for this kind of situation is the simulation of a persistent disease like tuberculosis, whose description requires models to run over decades [12]. However, the description of short-cycle diseases might require the usage of M3 instead of M2 too whenever calibration is not an option and the infectiousness of the pathogen is to be accepted from an a-priori source.

Summing up, using each of the different methods here described can result into significantly different projected contact patterns and modelers should be aware of the implications that this has on disease modelling. To illustrate such implications, in the next section we explore the quantitative implications of using each of the methods discussed here, by comparing the contact-structures themselves and simulating epidemic phenomena where contacts are described according to each of them.

## Results

### Reciprocity error

In order to study the error incurred when using M0, we transform the contact matrices obtained from empirical studies in different geographic settings to new matrices that correspond to the same location but at different years (that could be past records or future projections). As the population changes over time, the new matrices incorporate the population demographies of the same setting across time. We define the reciprocity error as the coefficient of variation of the number of contacts measured in both directions, which gives us a matrix that we will call non-reciprocity matrix (*NR*_*i*,*j*_). It is an antisymmetric matrix, in which a positive value of the entry (*i*, *j*) means that there are more contacts from *i* to *j* than in the opposite direction, and viceversa. A value of 0 would mean that the contacts between *i* and *j* are well balanced. More details can be found in the Supplementary Information.

**Fig 1:**
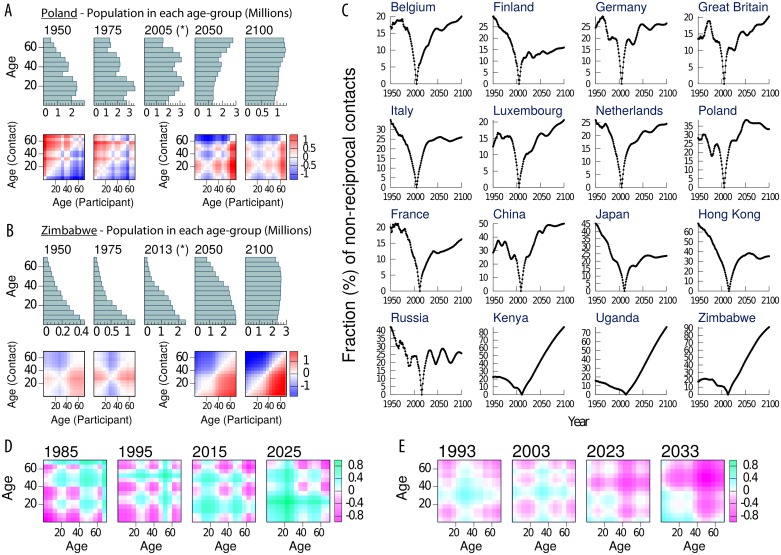
Analysis of methods M0 and M1. A-B: Demographic structures for different years and the respective non-reciprocal matrices $NR_{i,j}^{\prime }$ for Poland and Zimbabwe respective using M0. C: Evolution of the total fraction of non-reciprocal contacts for M0 in the 16 geographic settings analyzed in this study. D-E: $\log\;2(\frac{\Gamma_{i,j}^{\prime }}{\Gamma_{i,j}})$ for Poland and Zimbabwe respectively, in four different years (10/20 years before/after the measurement of the contact patterns) for M1. The original data corresponds to 2005 for Poland and 2013 for Zimbabwe.

In Fig 1 we represent the demographic structures of Poland (panel A) and Zimbabwe (panel B) for different years alongside the corresponding non-reciprocity matrices. In the case of European countries (Poland in panel A as an example), demographic structures have suffered from an ageing process during the last decades, which is predicted to continue in the future. This ageing tends to provoke negative values under the diagonal for the matrices *NR*_*i*,*j*_ when we assumed past demographic structures, while the opposite will occur in the future. The behaviour for African countries (Zimbabwe in panel B) is slightly different, as their demographies have been more stable for the last decades and only now they are beginning to age faster. In brief, when we use directly a contact pattern in a demographic structure that is younger than when it was measured, it will lead to an overestimation of the contact rate of (and the force of infection corresponding to) the youngest age-groups. The opposite will occur when we use contact patterns in an older population.

In Fig 1C we represent the evolution of the proportion of non-reciprocal contacts for all 16 geographic settings studied here (see Supplementary Information). This magnitude is equal to zero in the year when the contact matrix was measured, as we have applied a correction for the empirical matrices to fulfill reciprocity at the reference case. However, it dramatically increases as we move far from the year of the survey. In the examples shown here, only two years before/after the survey time, the fraction of non-reciprocal contacts already reaches 5%. Note that methods M1, M2 and M3 are well balanced by construction, thus *NR*_*i*,*j*_ = 0 for every (*i*, *j*) when using any of them.

### Intrinsic connectivity error

We next study the evolution of the ratio between the age-dependent contact rates and an homogeneous mixing scenario. This ratio gives us the matrix Γ_*i*,*j*_, defined as the intrinsic connectivity in Eq 4. The entries of Γ_*i*,*j*_ are bigger than 1 when the interactions between age-groups *i* and *j* surpasses what it is expected from the case of homogeneous mixing, and smaller than 1 in the opposite case. See the Supplementary Information for more details.

In Fig 1D and 1E we show 4 snapshots of the ratio of the intrinsic connectivity and the original survey ($\Gamma_{i,j}^{\prime }/\Gamma_{i,j}$) obtained using M1 for Poland and Zimbabwe respectively. Each panel corresponds to an adaptation of the contact matrix to the population demography of the countries 10 and 20 years before and after the survey (i.e., the 4 matrices correspond to *t* = *τ* − 20*y*, *t* = *τ* − 10*y*, *t* = *τ* + 10*y* and *t* = *τ* + 20*y*). We can see that, even if M1 corrects the appearance of non-reciprocity, this method changes the tendency of some age-groups to mix with respect to others. Specifically, we can see that M1 will over-represent contacts between young individuals (and under-represent contacts between old individuals) as the population gets older.

Furthermore, the previous results are quantitatively important. For instance, if we were to use the contact matrices that we have from Poland (measured in 2005) today (2018), we would have that the ratio $\Gamma_{i,j}^{\prime }/\Gamma_{i,j}$ surpasses 1.5 for some specific age-group pairs, while it goes down to almost 0.5 in others, or, in other words, the usage of M1, which does not take into account the changes in the fractions of individuals in each age-strata that occurred between 2005 and 2018, causes a bias of more than 50% in the contact densities projected between certain age groups. Consequently we say that M1 does not preserve intrinsic connectivity. The density correction (M2) avoids this problem, as it explicitly considers a fixed intrinsic connectivity matrix (Γ_*i*,*j*_ as defined in the Methods section) that is modified according to the density of each age-group (see Eq 3).

### Evolution of mean connectivity

**Fig 2:**
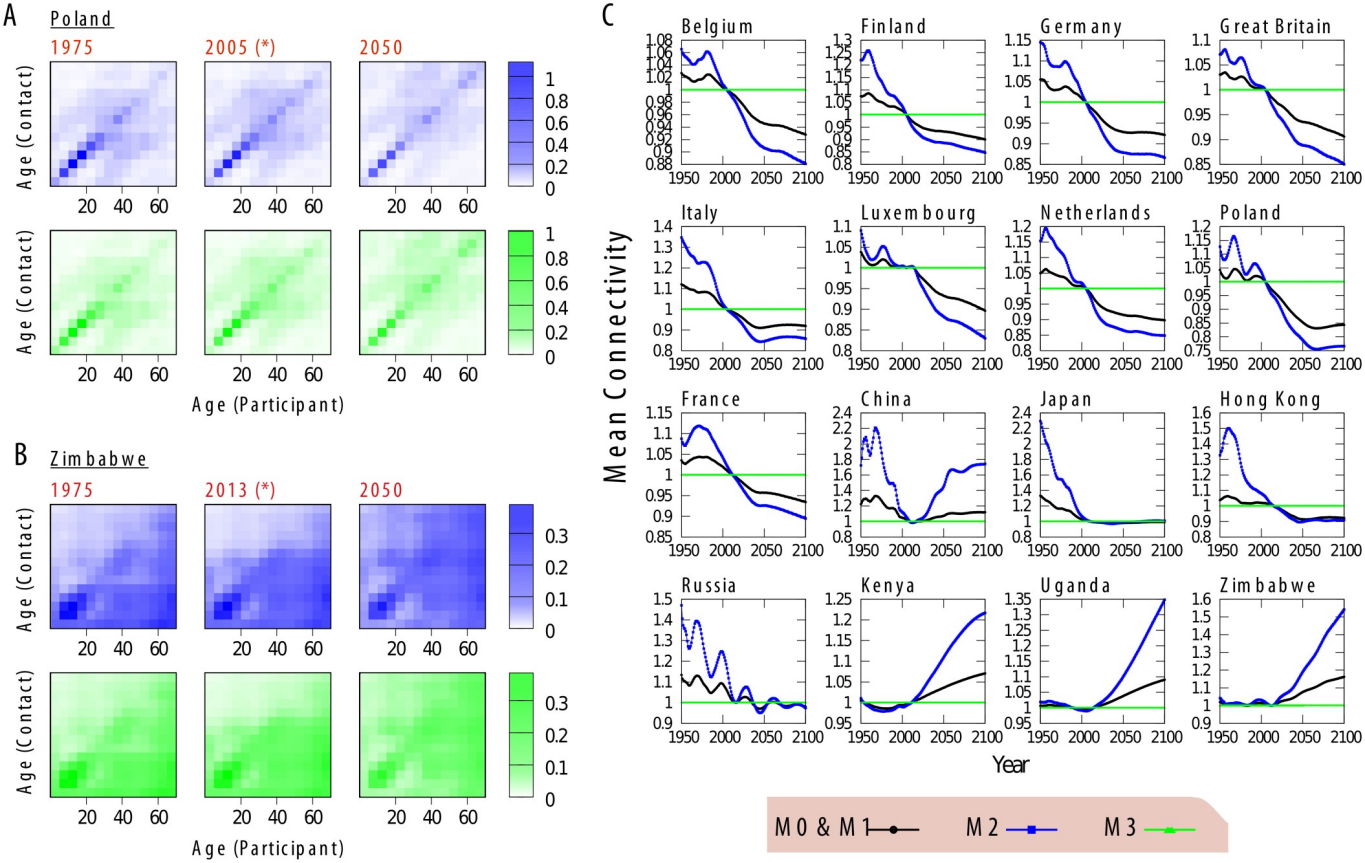
Analysis of methods M2 and M3. A-B: Contact patterns *M*_*i*,*j*_(*t*) for five different years with methods M2 (blue) and M3 (green) for Poland and Zimbabwe, respectively. C: Evolution of Mean Connectivity for M2 (blue), M3 (green) and M0 and M1 (black, both methods give the same mean connectivity).

In Fig 2A and 2B we represent the contact patterns obtained with M2 and M3 for Poland and Zimbabwe, respectively, in different years. We see how, specially in the case for Zimbabwe, as the population gets older, the values of the matrix below the diagonal (contacts toward young individuals) fade in favor of contacts toward older individuals as those age-groups gain more representation. As for the mean connectivity (Fig 2C), considering the evolution of contact patterns in M2 or considering them constant (M0) leads to the same qualitatively behaviour, although variances are higher with M2. These trends are decreasing in Europe and increasing in Africa. M0 and M1 have the same mean connectivity, as M1 consists basically of a rewiring of those connections that exist in M0 in order to correct for reciprocity. M3 is a normalization of M2 so the connectivity is constant in this case.

### Geographical comparisons

**Fig 3:**
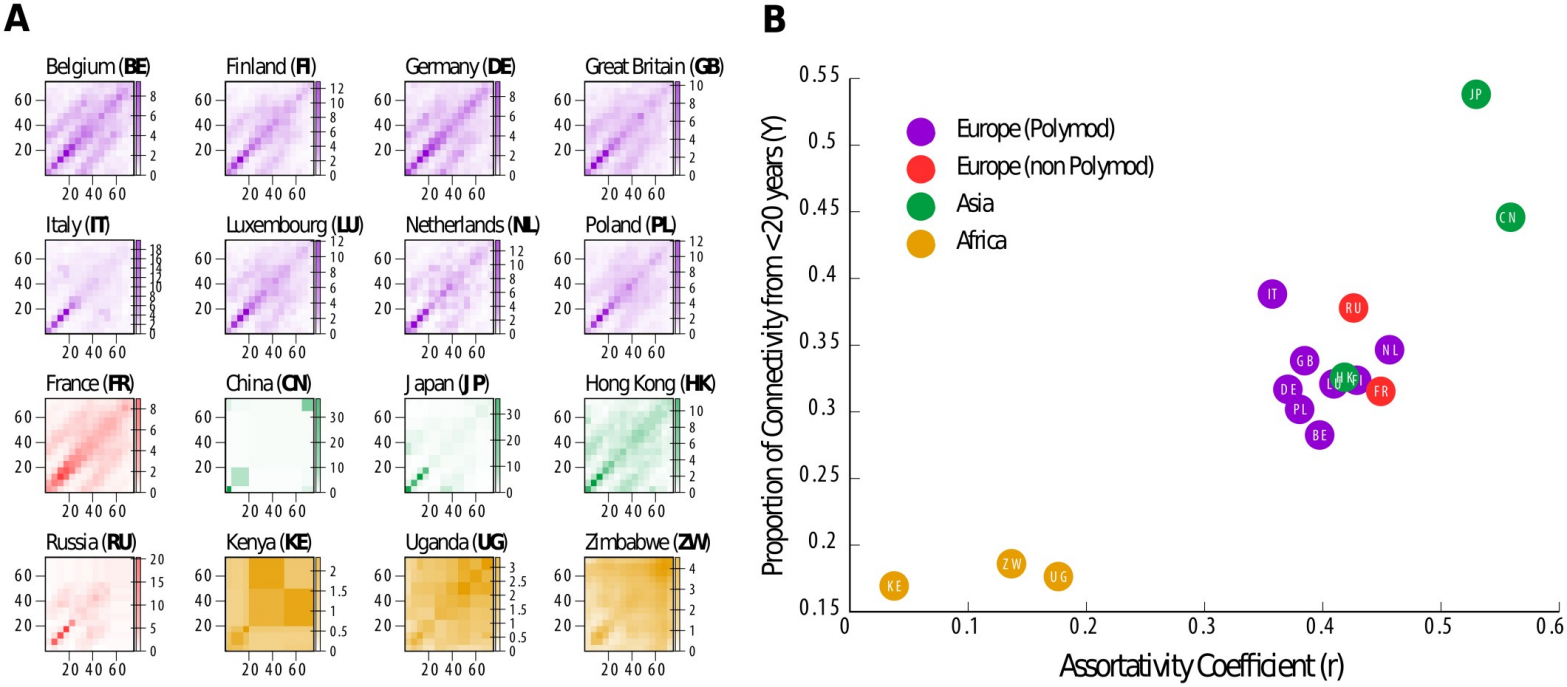
Geographical comparison of empirical contact matrices. A: Γ_*i*,*j*_ matrices for the 16 geographic settings considered in this work. B: Proportion of the overall connectivity that comes from individual with less than 20 years (Y) vs the assortativity coefficient (r) for the 16 settings.

The intrinsic connectivity matrices Γ_*i*,*j*_ that we obtain for every country allow us to compare the contact patterns of different settings once the influence of demography has been accounted for, and removed. In Fig 3A we represent these matrices for the 16 geographic settings analyzed in this work. Just by visual inspection we can identify some distinctive features: European matrices are more assortative and present higher interaction intensities among young individuals than African ones. To formalize this observation, in Fig 3B, we place the different matrices in a two dimensional plot defined by the proportion of overall connectivity produced by young individuals and the assortativity coefficient (see Supplementary Information for the definition of these quantities). African and European countries cluster around different values of these two magnitudes: specifically, in African countries we found less assortativity and the contacts are less dominated by young individuals than in the European countries. As for the Asia region we see that Japan and China have significantly higher assortativity and fraction of contacts among young individuals than either African or European countries. In turn, Hong Kong, with its particular geographic idiosyncrasy- a special administrative region, predominantly urban, with one of the highest population densities in the world-, presents an intrinsic connectivity matrix that is more similar to one from a European country than from China or Japan.

### Short cycle SEIR dynamics

Up to now, we have shown that there are several ways to deal with demographic change and evolving populations regarding the structure of the contact patterns for a given population. We next address how these different methods impact disease modeling. To this end, we implement a Short cycle SEIR model (details can be found in the Supplementary Information) to study a situation where a short-cycle, influenza-like pathogen appears in a given location in subsequent times. We consider two different modelling scenarios. In scenario 1 the pathogen infectiousness is independently calculated in each outbreak to ensure that all outbreaks have the same reproductive numbers independently of the eventual changes in contact matrices. By doing this, we aim at simulating a situation where a pathogen appears recurrently on a population, and its modelling relies on independent calibration of each outbreak. Then, in scenario 2, we model a situation when independent outbreak recalibration is not possible (or pertinent), and the infectiousness is assumed to be known (and constant) in all outbreaks. Under these hypothetical scenarios, we would like to know how different would be the predicted size of the epidemic as a result of considering different contact matrices coming from the different projection methods proposed in this work. In particular, scenario 1 is instrumental to distinguish the outcomes from models M0 and M1 from either M2 or M3. However, in this case the infectiousness is recalibrated in each event to ensure that all outbreaks have the same reproductive numbers. As a consequence, since the contact matrices derived from M2 and M3 only differ by a global scaling factor, the recalibration procedure absorbs the differences between M2 and M3, making them indistinguishable. In turn, scenario 2 simulates a situation where the election between M2 or M3 becomes of central relevance, since the basic reproductive number of outbreaks will now depend on the contacts produced by each method. These two scenarios are designed to recapitulate the two paradigmatic modeling situations discussed in the Methods overview section: the case where a short outbreak of a relatively unknown pathogen has to be modelled upon infectiousness calibration (scenario 1: emergent pathogens, influenza, etc.) versus the case where calibration is not an option, or model simulations extend in time (scenario 2: persistent diseases and/or a-priori known pathogens).

**Fig 4:**
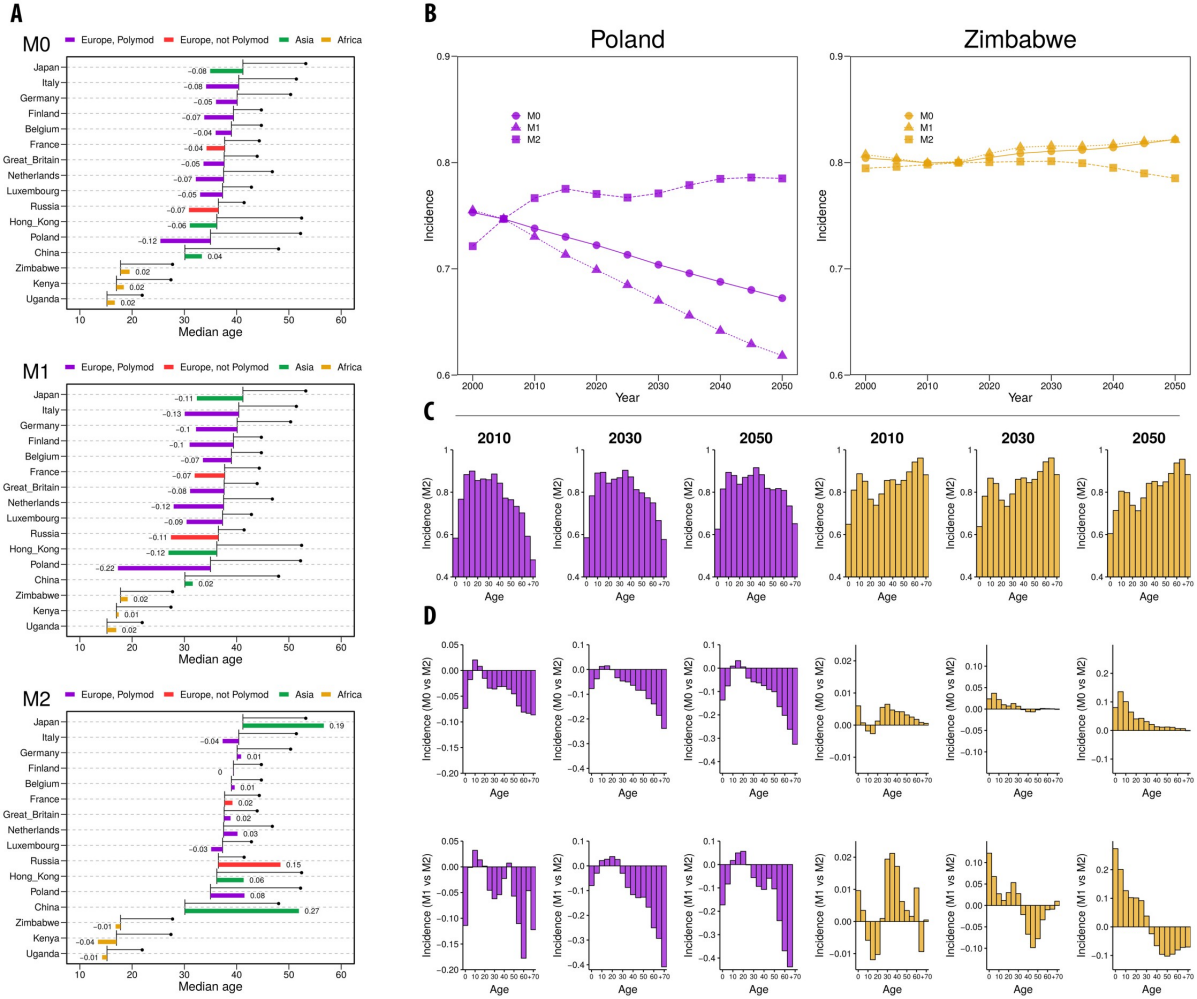
SEIR dynamics (scenario 1). A: Median age at 2000 and 2050 (black line, beginning with the value at 2000 and ending with a bullet point with the value at 2050) for the 16 geographic settings considered and relative variation in incidence over the same period (colored bars), for M0, M1 and M2. B: Incidence (over all ages) vs Year for Poland (purple) and Zimbabwe (orange) using M0, M1 and M2/M3. C: Incidence by age group for Poland and Zimbabwe in 2010, 2030 and 2050 using M2. D: Relative differences of the incidence by age group of M0 and M1 with respect to M2 (or M3) ($\frac{Inc(M0)-Inc(M2)}{Inc(M2)}$ and $\frac{Inc(M1)-Inc(M2)}{Inc(M2)}$).

**Fig 5:**
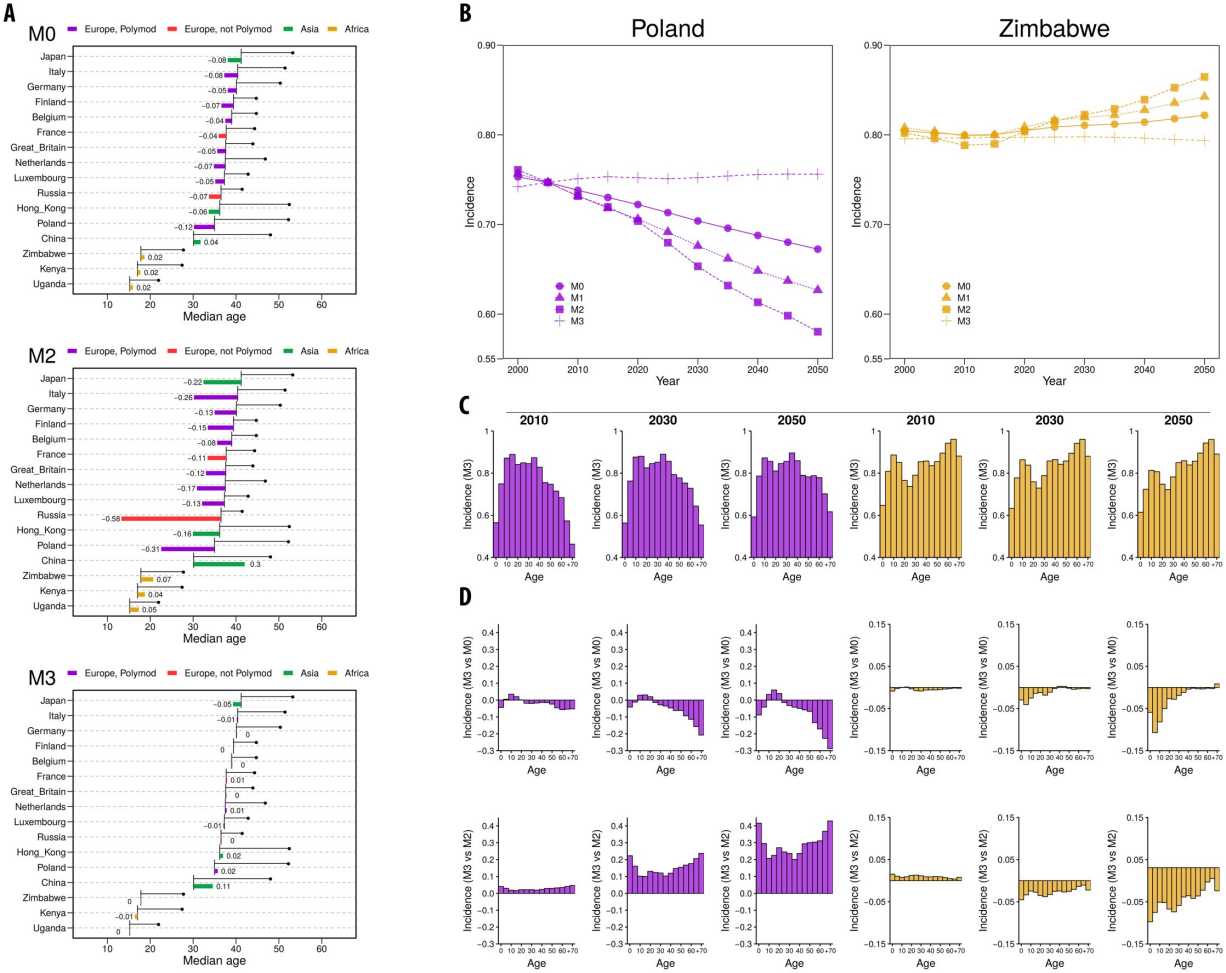
SEIR dynamics (scenario 2). A: Median age at 2000 and 2050 (black line, beginning with the value at 2000 and ending with a bullet point with the value at 2050) for the 16 geographic settings considered and relative variation in incidence over the same period (colored bars), for M0, M2 and M3. B: Incidence (over all ages) vs Year for Poland (purple) and Zimbabwe (orange) using M0, M2 and M3. C: Incidence by age group for Poland and Zimbabwe in 2010, 2030 and 2050 using M3. D: Relative differences of the incidence by age group of M0 and M2 with respect to M3 ($\frac{Inc(M0)-Inc(M3)}{Inc(M3)}$ and $\frac{Inc(M1)-Inc(M3)}{Inc(M3)}$).

The results of this exercise are presented in Fig 4 (scenario 1) and Fig 5 (scenario 2). Regarding scenario 1, in Fig 4, panel A we can see that, while methods M0 and M1 predict lower age-aggregated incidences in European countries in 2050 with respect to 2000, M2 reduces these differences and the incidences are comparable for both years or even positive (M3 is not included here, for it would produce exactly the same results of M2). A different situation is observed in Africa, where M0 and M1 predict an increase in incidence in the future while using M2 would lead to a decrease, though differences remain small (less than 5% of variation).

In panel Fig 4B we represent, for two examples of Europe and Africa (Poland in purple and Zimbabwe in orange), the temporal evolution of the incidence observed with the different methods. Furthermore, we represent the age-specific incidence for both countries in three different years: 2010, 2030 and 2050 (Panel Fig 4C). The age-distribution of the incidence evidences the differences in connectivity patterns between Poland and Zimbabwe. While the incidence in elderly people drops in Poland (as the contact rates for those age-groups also drop), it remains high in Zimbabwe for the same age-groups.

The different methods of implementing contact rates also affect the age-specific incidence. In panel Fig 4D we represent the relative variation in age-specific incidence obtained with methods M0 and M1 with respect to M2 for Poland and Zimbabwe. In Poland we see that M0 and M1 tend to underestimate the incidence specially among the elder age-groups. In Zimbabwe M0 tends to overestimate the incidence among young individuals, while with M1 we encounter both effects: and overestimation among the youngest and a underrepresentation among the eldest.

The reshaping of the age-specific incidence between models is coherent with the changes in topology already studied. For the case of M0, i.e., maintaining the contact patterns constant in time, we have that in the future, as the demographic structure shifts to older populations, contacts toward children will be overrepresented and contacts toward adults will be underrepresented. At first order we can obviate the contacts that are far from the diagonal, and establish that M0 mainly underrepresents contacts between adults and overrepresents contacts between young individuals (in the context of aging populations). Thus, we will obtain an underrepresentation of the incidence in adults, and the opposite in children. However, as the eldest age-groups increase their population in Europe, they dominate the dynamics and cause and underestimation of the global incidence that eventually affects all age-groups. In African countries, where the contact patterns are less assortative than European countries, this effect is smaller. Besides, as African populations are still young even in 2050, the overestimation of young contacts dominates the dynamics, and the differences in incidence are mainly positive. The situation is similar for M1. As represented in Fig 1D and 1E, for M1 we also have an underrepresentation of contacts between adults and an overestimation between young individuals, yielding to similar results to M0.

In scenario 1, where the infectiousness *β* is recalibrated in each outbreak, the mean connectivity does not play a role in the size of the outbreak. Thus M2 and M3 lead to the same outbreaks’ description, with the exception of the inferred values of *β* needed to produce them, which would contribute, nonetheless, to different evaluations of the epidemiological risk. This dynamical equivalence emanates only from the assumption that reproductive numbers can be measured at the early stages of any of the epidemics being simulated in each year, which is a conservative -often optimistic- assumption. However, in the alternative scenario where no initial calibration is possible or prescribed, and constant infectiousness values are accepted through all possible times, the equivalence between M2 and M3 is broken (scenario 2, shown in Fig 5). As discussed in the methods overview section, this is conceptually similar to the task of producing long term forecasts of persistent diseases [12], based on epidemiological parameters calibrated on an initial time-window.

As we show in Fig 5, when we do not recalibrate the infectiousness, M2 and M3 show a very different behaviour. While M3 leads to an outbreak size that is essentially invariant in time -due to stochasticity-, the outcome predicted from M2 is highly variable. Specifically, we see how European countries produce outbreak sizes that decrease in time while the opposite occurs for African countries, which matches the evolution of the mean connectivity as shown in Fig 2C. Regarding the age distribution of the incidence under M3 (Fig 5C), we see a similar pattern to the one seen in scenario 1. The comparison of the age distributions from methods M2 and M3 (Fig 5D) shows that the differences between both methods, already discussed at the aggregated level, also occur in the same direction within all age groups.

All together, these results illustrate how a poor adaptation of the contact patterns observed in the past in a given country to a later time point can translate into epidemiological forecasts that are highly biased. On the one hand, we have seen how the limitations of M0 and M1 at describing reciprocity and intrinsic connectivity patterns translate into inconsistent results. On the other hand, regarding the comparison between the two methods based on the density correction for available contactees -M2 and M3-, we have seen how the introduction of a normalization term in M3 aimed at preserving the overall connectivity is specially relevant in the cases where epidemiological parameters cannot be calibrated at the early stages of the epidemic phenomena to be modelled.

## Discussion

Summarizing, empirical contact patterns belong to a specific time and place. If we want to integrate the heterogeneity of social mixing into more realistic models, we need to address how (and in what cases) to export contact patterns from empirical studies to the populations we want to study. In this work, we have studied and quantified the significant bias incurred when a specific contact pattern is blindly extrapolated to the future (or the past), even if we remained inside the same country where those contacts were measured. In fact, only a couple of years after the measurement of these contact patterns, the changes in the age structure of the population make them vary significantly. Thus, for any meaningful epidemic forecast based on a model containing age-mixing contact matrices, we would need to adapt them taking into account the evolution of the demographic structures. Moreover, as we have shown, even for cases that do not expand into long periods of time and a constant demography could be assumed, it is necessary to make an initial adaptation of whatever empirical contact structure we want to implement, into the specific demographic structure of our system. We have also seen how these relevant differences in the topology of contacts yield to significant consequences for the spreading of a disease. Applying different methods to deal with contact patterns leads to important differences not only in the global incidence for a SEIR model, but also on age-specific incidences. Having such an important impact for the spreading of a disease, the insights provided by this work should be taken into consideration by modelers and also by public health decision-makers.

In a similar way, we have explored the differences between the contact patterns of different geographic settings. Thus, we have found the existence of some specific characteristics beyond the underlying demographic pyramids, which warns against exporting contact patterns across different geographic areas (i.e. continents). Since there are different intrinsic connectivity patterns (i.e., once demography effects have been subtracted) across countries, it is also likely that there exists a time-evolution of the intrinsic connectivity inside the same setting. Although it is impossible to predict how society will change in the future, we should always take this into account as a limitation in any forecast for which the heterogeneity in social mixing is a key element.

Finally, we note that there are some limitations that could affect quantitatively the results shown in this work. First of all, we have derived the contact patterns of the different studies according to the demographic structures of the specific country for the year the survey took place. Thus, we are implicitly assuming that the setting where the different surveys were performed are comparable with the national data in terms of their demographic pyramids. In other words, we assume that the surveys are representative of the population at large. This is likely true for most of the geographic settings analyzed, but there are certain cases in which this might not be the case, either because of small study size or putatively biased recruitment of participants. Besides, as we have already discussed in the Methods section, the different granularity (i.e., definition of the age-groups) used throughout the bibliography studied also imposes some limitations when comparing the data. It is also worth pointing out that, although in this work we have focused on age-structured systems (which has had its relevance in recent history of epidemiology), the problem studied here can be extrapolated to other models that might categorize their individuals based on other different traits that determine their social behavior.

The results reported here and their implications open several paths for future research. One is related to the social mixing patterns themselves. In order to predict the large-scale spreading of a disease, multiple scales need to be integrated and coupled together. This implies that when integrating different spatial scales, we need to deal with different contact matrices and local demographies. For instance, in developed countries, it is known that the structure of the population is not the same in the most central or most populated cities as compared to smaller ones or the countryside. Thus, nation-wide demographies and surveys to infer contact matrices might need to be disaggregated. What is the right spatial scale to measure both quantities is an interesting and unsolved question. In this sense, here we have limited our simulated disease scenario to the case of isolated populations, but it remains to be seen what are the effects over a meta-population framework, in which we have mobility between sub-populations of potentially very different demographic structures. We plan to explore these issues in the future.

## Supporting information

S1 Supporting information Extended details on methods and additional analyses. (PDF)
