# Supplementary material for: Projecting social contact matrices to different demographic structures
Source: PLoS Comput Biol. 2018 Dec 7;14(12):e1006638. doi: 10.1371/journal.pcbi.1006638 (PMC6300299; doi:10.1371/journal.pcbi.1006638)
Supplement: S1 Supporting information — (PDF) [file pcbi.1006638.s001.pdf]

Projecting social contact matrices to different demographic structures.
Supplementary Information

Sergio Arregui[a,b], Alberto Aleta[a,b], Joaquín Sanz[c,d,‡], and Yamir Moreno[a,b,e,‡]

[a]Institute for Biocomputation and Physics of Complex Systems (BIFI), University of Zaragoza, Spain
[b]Department of Theoretical Physics, University of Zaragoza, Spain
[c]Department of Genetics, Saint-Justine Hospital Research Center, Montreal, Canada
[d]Department of Biochemistry,University of Montreal, Canada
[e]ISI Foundation, Turin, Italy

‡These authors contributed equally to this work.

1 Methods

1.1 Different Scales to define Contact Patterns

Although the contact matrices are often described with the matrix $M_{i,j}$ that we define in the main text (which we will call the intensive scale), sometimes two other scales are used:

- Extensive scale: $C_{i,j}$ that is the total number of contacts between age-groups i and j.

$$C_{i,j} = M_{i,j} N_i \tag{1}$$

- Density scale: $F_{i,j}$ that is the fraction of contacts between age-groups i and j among all possible contacts that actually take place.

$$F_{i,j} = \frac{C_{i,j}}{N_i N_j} = \frac{M_{i,j}}{N_j} \tag{2}$$

These two scales are symmetric by construction. Therefore they might seem a better option to work with, as they directly avoid the problem of non-reciprocity. However, unlike $M_{i,j}$ they do not remain invariant when the total number of individuals increases maintaining the shape of the demographic structure, so a correction for the total population will always be needed. In this work, we describe magnitudes in the intensive scale mostly, but the translation to other scales is always possible through equations 1 and 2. It is also possible to work with any of these scales as long as the force of infection is properly defined.

1.2 Treatment of empirical survey matrices

As stated in the main text, we need to perform some transformations before we can compare the different empirical studies:

- Most of the studies report the matrix in the intensive scale ($M_{i,j}$). Some of these studies however (specifically France[1], Japan[2] and China[3]) report their results using the density scale (see equation 2) except for a global factor. Thus, we transform them so they operate in the scale of $M_{i,j}$, assuming the demographic structure as reported by the United Nations (UN) Population Database[4] for the specific year of the survey.
- The studies use different age granularities. We adapt all surveys to the division of age groups used by the POLYMOD project, i.e., 15 age groups: 0-5, 5-10, 10-15,..., 65-70, 70+. For this adaptation sometimes we have to aggregate age-groups (some studies have more divisions for young children) but mostly we have to divide age-groups. In order to do so, we assume homogeneity inside the broader original age-groups. The studies with less age-groups (China that has 4, and Kenya that has 6) are therefore more sensitive to this process.

In Table A we summarize the information about the 16 empirical contact matrices collected for this work.

**Country**	**Year**	**Age Groups**	**Participants**	**Reference**
Belgium	May 2005 - September 2006 (2005)	15: 0-5, 5-10,...,65-70,70+	750	Mossong et al. (POLYMOD)[5]
Finland	May 2005 - September 2006 (2005)	15: 0-5, 5-10,...,65-70,70+	1006	Mossong et al. (POLYMOD)[5]
Germany	May 2005 - September 2006 (2005)	15: 0-5, 5-10,...,65-70,70+	1341	Mossong et al. (POLYMOD)[5]
Great Britain	May 2005 - September 2006 (2005)	15: 0-5, 5-10,...,65-70,70+	1012	Mossong et al. (POLYMOD)[5]
Italy	May 2005 - September 2006 (2005)	15: 0-5, 5-10,...,65-70,70+	849	Mossong et al. (POLYMOD)[5]
Luxembourg	May 2005 - September 2006 (2005)	15: 0-5, 5-10,...,65-70,70+	1051	Mossong et al. (POLYMOD)[5]
Netherlands	May 2005 - September 2006 (2005)	15: 0-5, 5-10,...,65-70,70+	269	Mossong et al. (POLYMOD)[5]
Poland	May 2005 - September 2006 (2005)	15: 0-5, 5-10,...,65-70,70+	1012	Mossong et al. (POLYMOD)[5]
France	February - May 2012	15: 0-5,5-10,...,65-70,+70	2033	Béraud et al.[1]
Russia	January - February 2016	12: 0-5,5-10,...,50-55,+55	505	Ajelli and Litvinova[6]
China	2009 - 2010 (2009)	4: 0-5,6-19,20-64,+65	1821	Read et al.[3]
Japan	Spring 2011	12: 0-2,3-5,6-11,12-14,15-19,20-29,...,70-79,+80	3146	Ibuka et al.[2]
Hong Kong	2015-2016 (2015)	15: 0-5,5-10,...,65-70,+70	1149	Leung et al.[7]
Kenya	August 2011 - January 2012(2011)	6: 0-1, 1-5, 6-15, 16-19, 20-49, +50	1080	Kiti et al.[8]
Uganda	January - March 2014	10: 0-2, 2-4,5-9, 10-14, 15-24,...,55-64,+65	568	Le Polain de Waroux et al.[9]
Zimbabwe	March - August 2013	16: 0-5,5-10,...,70-75,75-80	2490	Melegaro et al.[10]

Table A: Information about the 16 contact matrices collected. When the period of the study comprises more than one year, we specify in parenthesis the year taken as reference for posterior analysis.

From each one of these studies, and after performing the transformations previously specified (when needed) we obtain a contact matrix $M_{i,j}^{(surv.)}$. However, due to limitations in the survey and errors in the reporting process, these matrices will not fulfil reciprocity perfectly. Thus, we perform an initial correction for reciprocity, that consists of averaging the number of contacts measured in one direction (i to j) and the reciprocal (j to i), which for the C matrix means:

$$C_{i,j}^{(surv.)} = \frac{M_{i,j}^{(surv.)} N_i n_i + M_{j,i}^{(surv.)} N_j n_j}{n_i + n_j} \tag{3}$$

and, when taken back to the M scale:

$$M_{i,j}^{(rec.)} = \left(M_{i,j}^{(surv.)} n_i + M_{j,i}^{(surv.)} \frac{N_j}{N_i} n_j \right) \frac{1}{n_i + n_j} \tag{4}$$

where n_i is the number of participants in age-group i and N_i is the population of age-group i in the corresponding country during the specific year of the survey as extracted from the UN database[4]. Thus, we are applying a weighted average that needs the age-distribution of the participants. When the latter information is not available, we perform a regular average. This process can also induce some bias since for some cases, the survey of contacts has been performed in some specific settings (rural or semi-urban) that might not be representative of the whole country. This correction of reciprocity has been used before, either to present empirical data[10] or to ensure reciprocity in a simulation by adapting the contact patterns to the specific demographic structure considered.[11,12] The possibility to use this pair-wise correction, which we will label as Method 1 (M1) to solve the problem of reciprocity has been explored in the main text.

In equation 4, we have named the resultant matrix $M_{i,j}^{(rec.)}$, where the super-index indicates that the matrix fulfils reciprocity. We still apply one more transformation, that consists of normalizing the matrices so that the mean connectivity of the population is equal to 1. The mean connectivity follows the formula:

$$\langle k \rangle = \frac{\sum_{i,j} M_{i,j}^{(rec.)} N_i}{N} \tag{5}$$

where $N = \sum_i N_i$. Thus, we obtain:

$$M_{i,j}^{(rec.+norm.)} = \frac{M_{i,j}^{(rec.)}}{\langle k \rangle} = \frac{M_{i,j}^{(rec.)} N}{\sum_{i,j} M_{i,j}^{(rec.)} N_i} \tag{6}$$

Hence, in this work we are not addressing the average intensity of contacts (and how it differs between countries) and we focus exclusively on the relative differences between age-groups. Although geographical differences in global contact rates is a problem of interest, we have not studied them for three main reasons. First, for France, Japan and China, the studies report a matrix that is proportional to $F_{i,j}$ and we do not have direct

information on the intensity of contacts. Second, it depends on the definition of contact itself and therefore methodological biases could arise. Most studies define a contact as any interaction that includes physical touching and/or face-to-face conversation for a minimal time period (that varies between studies), and even report data on physical contact separatedly, but some only consider one type of contact (only physical contacts in Kenya[8], and only conversation in Russia[6] and Uganda[9]). And third, for many problems, the scaling of this contact matrix is performed independently and therefore not having the overall intensity of contacts is irrelevant.[13]

After this process we end up with the matrix $M_{i,j}^{(rec.+norm.)}$ (that we will name simply as $M_{i,j}$) for each one of the 16 countries for which we have contact data. We assume that, except for the limitations in the survey, these matrices are valid in their respective countries during the year when they were measured.

1.3 Properties of projection methods

The different methods of transforming a matrix $M_{i,j}$ into a matrix $M'_{i,j}$ when moving from a demography N_i to a demography N'_i that we have studied can be formulated as:

- M0: Unadapted Contact Patterns.

$$M'_{i,j} = M_{i,j}. \tag{7}$$

- M1: Pair-wise Correction

$$M'_{i,j} = \frac{1}{N'_i}\frac{1}{2}\left(M_{i,j}N'_i + M_{j,i}N'_j\right) = M_{i,j}\frac{1}{2}\left(1 + \frac{N_iN'_j}{N_jN'_i}\right). \tag{8}$$

- M2: Density Correction

$$M'_{i,j} = M_{i,j}\frac{NN'_j}{N_jN'}. \tag{9}$$

- M3: Density Correction + Normalization

$$M'_{i,j} = M_{i,j}\frac{N'_j}{N_j}\frac{N'}{\sum\limits_{i,j} M_{i,j}\frac{N'_iN'_j}{N_j}}. \tag{10}$$

1.3.1 Reciprocity

The main property that a Contact Matrix should fulfill is reciprocity in the number of contacts:

$$M_{i,j}N_i = M_{j,i}N_j \Longrightarrow \frac{M_{i,j}}{M_{j,i}} = \frac{N_j}{N_i}. \tag{11}$$

Thus, when moving to a different demographic structure (where, in general, the ratio $\frac{N_j}{N_i}$ will change), M0 does not preserve reciprocity:

$$\frac{M'_{i,j}}{M'_{j,i}} = \frac{M_{i,j}}{M_{j,i}} = \frac{N_j}{N_i} \neq \frac{N'_j}{N'_i}. \tag{12}$$

However, it is easy to see that the rest of the methods do preserve reciprocity:

- Reciprocity in M1:

$$\frac{M'_{i,j}}{M'_{j,i}} = \frac{M_{i,j}}{M_{j,i}}\frac{1+\frac{N_iN'_j}{N_jN'_i}}{1+\frac{N_jN'_i}{N_iN'_j}} = \frac{N_j}{N_i}\frac{\frac{N_jN'_i+N_iN'_j}{N_jN'_i}}{\frac{N_iN'_j+N_jN'_i}{N_iN'_j}} = \frac{N'_j}{N'_i}\frac{N_jN'_i+N_iN'_j}{N_iN'_j+N_jN'_i} = \frac{N'_j}{N'_i}. \tag{13}$$

- Reciprocity in M2:

$$\frac{M'_{i,j}}{M'_{j,i}} = \frac{M_{i,j}}{M_{j,i}}\frac{NN'_j}{N'N_j}\frac{N'N_i}{NN'_i} = \frac{N_j}{N_i}\frac{N'_j}{N_j}\frac{N_i}{N'_i} = \frac{N'_j}{N'_i}. \tag{14}$$

- Reciprocity in M3:
$$\frac{M'_{i,j}}{M'_{j,i}} = \frac{M_{i,j}}{M_{j,i}} \frac{N'_j}{N'_i} \frac{N_i}{N_j} = \frac{N_j}{N_i} \frac{N'_j}{N'_i} \frac{N_i}{N_j} = \frac{N'_j}{N'_i}. \tag{15}$$
Notice that in this case the factor $\frac{N'}{\sum_{i,j} M_{i,j} \frac{N'_i N'_j}{N_j}}$ is the same for $M'_{i,j}$ and for $M'_{j,i}$.

As a global measure of the reciprocity error ($E \in [0,1]$) we use the total fraction of non-reciprocal contacts:
$$E = \frac{\sum_{i,j>i} |C_{i,j} - C_{j,i}|}{\frac{1}{2}\sum_{i,j} C_{i,j}} = \frac{\sum_{i,j>i} |M_{i,j}N_i - M_{j,i}N_j|}{\frac{1}{2}\sum_{i,j} M_{i,j}N_i}. \tag{16}$$
It will be zero for methods M1, M2 and M3.

1.3.2 Intrinsic Connectivity

Let us define the matrix $H_{i,j}$ as the contact pattern resulting after assuming homogeneous mixing (also known as proportional mixing) embedded in a population structure N_i (normalized to have a connectivity of 1):
$$H_{i,j} = \frac{N_j}{N}, \tag{17}$$
where $N = \sum_i N_i$.

Thus, we can define the intrinsic connectivity $\Gamma_{i,j}$ as the ratio between the contact rate and what we would have in the case of homogeneous mixing ($\Gamma_{i,j} = M_{i,j}/H_{i,j} = M_{i,j}\frac{N}{N_j}$). The entries of this matrix will be larger than 1 if the connectivity between age-groups i and j is more than what would be expected under the homogeneous mixing scenario, and viceversa.

We study the evolution of this matrix (i.e., $\frac{\Gamma'_{i,j}}{\Gamma_{i,j}} = \frac{M'_{i,j}}{M_{i,j}} \frac{N_j}{N'_j} \frac{N'}{N}$) with the four methods:

- Intrinsic Connectivity with M0:
$$\frac{\Gamma'_{i,j}}{\Gamma_{i,j}} = \frac{M'_{i,j}}{M_{i,j}} \frac{N_j}{N'_j} \frac{N'}{N} = \frac{N_j}{N'_j} \frac{N'}{N}. \tag{18}$$
It is not constant.

- Intrinsic Connectivity with M1:
$$\frac{\Gamma'_{i,j}}{\Gamma_{i,j}} = \frac{M'_{i,j}}{M_{i,j}} \frac{N_j}{N'_j} \frac{N'}{N} = \frac{1}{2}\left(1 + \frac{N_i N'_j}{N_j N'_i}\right) \frac{N_j}{N'_j} \frac{N'}{N} = \frac{1}{2}\left(\frac{N_j}{N'_j} + \frac{N_i}{N'_i}\right) \frac{N'}{N}. \tag{19}$$
It is not constant.

- Intrinsic Connectivity with M2:
It is constant by definition:
$$M_{i,j} = \Gamma_{i,j} \frac{N_j}{N} = \Gamma_{i,j} H_{i,j} \Longrightarrow \Gamma_{i,j} = \frac{M_{i,j}}{H_{i,j}} \text{ is constant.} \tag{20}$$

- Intrinsic Connectivity with M3:
$$\frac{\Gamma'_{i,j}}{\Gamma_{i,j}} = \frac{M'_{i,j}}{M_{i,j}} \frac{N_j}{N'_j} \frac{N'}{N} = \frac{N'_j}{N_j} \frac{N_j}{N'_j} \frac{N'}{\sum_{i,j} M_{i,j} \frac{N'_i N'_j}{N_j}} \frac{N'}{N} = \frac{N'}{\sum_{i,j} M_{i,j} \frac{N'_i N'_j}{N_j}} \frac{N'}{N}. \tag{21}$$
It only changes in a global factor $\frac{N'}{\sum_{i,j} M_{i,j} \frac{N'_i N'_j}{N_j}} \frac{N'}{N}$.

Only M2 (and M3 except for a global factor) preserves the intrinsic connectivity. M0 and M1 however, change the tendency of mixing between different age-groups. In order to understand the importance of this, let us suppose that we have an initial matrix that follows the assumption of homogeneity (i.e., $M_{i,j} = H_{i,j} \implies \Gamma_{i,j} = 1$). Then, we would have that from an initial situation of homogeneous mixing we end up with heterogeneities artificially produced by the change on the shape of the demographic pyramid if we follow methods M0 or M1, while M2 will still provide homogeneous mixing regardless of the demographic structure.

Also notice that there exists a relation between $\Gamma_{i,j}$ and the matrix $F_{i,j} = \frac{M_{i,j}}{N_j}$ that we described in equation 2. The method M2 can also be seen as the one that preserve the density scale applying a correction for the total population size $F'_{i,j} = F_{i,j}\frac{N}{N'}$.

1.3.3 Mean connectivity

The mean connectivity $\langle k \rangle$, i.e, the average number of contacts per individual, is given by:

$$\langle k \rangle = \frac{\sum_{i,j} M_{i,j} N_i}{N} \tag{22}$$

We have normalized the empirical contact matrices to have a mean connectivity of 1. When transferring these contact matrices to different demographies we have that the mean connectivity might change depending on the method:

- Mean connectivity in M0:

$$\langle k' \rangle = \frac{\sum_{i,j} M_{i,j} N'_i}{N'}. \tag{23}$$

- Mean connectivity in M1:

$$\langle k' \rangle = \frac{\sum_{i,j} M_{i,j} \frac{1}{2}\left(1 + \frac{N_i N'_j}{N_j N'_i}\right) N'_i}{N'} = \frac{\sum_{i,j} M_{i,j} N'_i}{N'}. \tag{24}$$

  As M1 consists of a re-arrengment of the contacts that already exist in M0 to correct reciprocity, it can be shown that they actually share the same mean connectivity.

- Mean connectivity in M2:

$$\langle k' \rangle = \frac{N}{(N')^2} \sum_{i,j} M_{i,j} \frac{N'_j N'_i}{N_j}. \tag{25}$$

- Mean connectivity in M3:

$$\langle k' \rangle = \frac{\sum_{i,j} M_{i,j} \frac{N'_j}{N_j} \frac{N'}{\sum_{i,j} M_{i,j} \frac{N'_i N'_j}{N_j}}}{N'} = \frac{\sum_{i,j} M_{i,j} \frac{N'_i N'_j}{N_j}}{\sum_{i,j} M_{i,j} \frac{N'_i N'_j}{N_j}} = 1. \tag{26}$$

  M3 consists of a normalization of M2, so it trivially preserves the mean connectivity.

1.4 Comparison of intrinsic contact patterns

With M2 and M3 we define the contact patterns as the product of two factors, the intrinsic connectivity $\Gamma_{i,j}$ and the density of available contactees. These matrices $\Gamma_{i,j}$ are proportional to the matrix of contacts that we will have in a perfectly squared demography (where all age-groups have the same size). Thus, the definition of the $\Gamma_{i,j}$ matrices is specially useful to compare the social mixing of different countries beyond the influence of demographic structure. We will define two magnitudes to characterize intrinsic contact patterns, the proportion of young (less than 20 years old) connectivity (Y) and the assortativity coefficient (r)[14].

$$Y = \frac{\sum_{i<20y.,j} \Gamma_{i,j}}{\sum_{i,j} \Gamma_{i,j}} = \frac{\sum_{i<20y.} k(i)}{\sum_i k(i)}, \tag{27}$$

$$r = \frac{\sum_{i,j}\left(\Gamma_{i,j} - \frac{k(i)k(j)}{\sum_{i,j}\Gamma_{i,j}}\right)E(i)E(j)}{\sum_{i,j}\left(k(i)\delta_{i,j} - \frac{k(i)k(j)}{\sum_{i,j}\Gamma_{i,j}}\right)E(i)E(j)}, \tag{28}$$

where $k(i) = \sum_j \Gamma_{i,j}$, and $E(i)$ is the (mean) age associated to age-group i.

1.5 SEIR model

To demonstrate the importance of choosing the correct procedure to project contact patterns in time, we simulate the spreading of an influenza like disease using the different methods described in this paper. We choose a short-cycle disease so that we can assume that the population structure is constant during each simulation. To parametrize our model we will use the values of an influenza outbreak that took place in Belgium in the season 2008/2009 [15].

The disease is described using a Susceptible-Exposed-Infectious-Recovered (SEIR) model. Susceptible individuals can catch the disease with a transmissibility rate β per-contact with an infective individual. Once infected, individuals remain on a latency state for $\epsilon^{-1} = 1.1$ days on average. Then, they become infectious for $\mu^{-1} = 3$ days on average, period when they can transmit the infection to susceptible individuals. After that, they recover and become immune to the disease. We use a discrete and stochastic model where the population is divided into 15 age classes. Social mixing is quantified by the contact matrices extracted from the data, normalized accordingly. Time is discretized with a time step of $\Delta t = 1$ day and the results are averaged over 10^4 runs.

We consider two different scenarios. For scenario 1, we consider the reproductive number to be $R = 2.12$, the same at all times and countries. With this value, we obtain the appropiate β for each country and each year so that the largest eigenvalue of the matrix

$$K_{i,j} = \frac{\beta}{\mu} M_{i,j} \frac{N_i}{N_j} \tag{29}$$

in the case of the methods M0, M1 and M3 and

$$K_{i,j} = \frac{\beta}{\mu} \Gamma_{i,j} \frac{N_i}{N} \tag{30}$$

in the case of the method M2 is equal to the reproductive number, R. Note that as we are adjusting β in each case to obtain the desired value of the reproductive number, the mean connectivity of the matrix will not play a role. Thus, we would obtain exactly the same results using M2 and M3, as their only difference is the global contact rate and it will be incorporated in β. For this reason, in the main text we have only compared the first three methods: M0, M1 and M2.

For scenario 2, we use the values of β obtained in 2005 at all different times. With a constant infectiousness, the differences in mean connectivity will change the outcomes, and M2 and M3 are not dynamically equivalent in this case.

2 Additional analysis

2.1 Comparison with Prem et al.

In a recent work by Prem et al. [16], the contact pattern from the Polymod project [5] (averaged over the 8 European countries) is exported to different countries. However, the approach proposed in [16] to complete that task implies the integration of a series of data of disparate nature: household structures, pupil-to-teacher ratios, school enrollment rates, and also age distributions. In this sense, although our work also allows to perform a similar analysis and project an averaged Polymod matrix (for example) to other non-Polymod countries, in our case, this can be done using demographic data on populations' age distributions alone without including further data. As a means to evaluate the accuracy of our more economic approach compared to that proposed in [16], we have focused on the eight non-Polymod countries for which we have independent empiric data (France, Russia, China, Hong Kong, Japan, Kenya, Uganda and Zimbabwe), and compared, for each of them, three different contact matrices: A) the empiric ones, B) the projections obtained starting from the Polymod data, and applying the

Conuntry	Our projection vs. Prem et al.	Prem et al. vs. Surveys	Our projection vs. Surveys
France	0.932	0.827	0.849
Russia	0.878	0.800	0.909
China	0.962	0.235	0.315
Japan	0.933	0.675	0.770
Hong Kong	0.909	0.661	0.856
Kenya	0.971	0.566	0.563
Uganda	0.948	0.649	0.670
Zimbabwe	0.964	0.573	0.609

Table B: Correlation index of the comparison of different contact patterns. For the 8 non-Polymod countries considered in this work, we make three comparisons: (1) our projections of the Polymod matrix vs. the projections made by Prem et al., (2) the projections made by Prem et al. vs the empirical data measured in those countries and (3) our projection vs the same empirical data.

transformations proposed in[16] and C) an equivalent projection of the Polymod pattern using either M2 or M3 as proposed in this work.

In order to obtain the projections based on our method, we first need to produce an average of the Polymod contact matrices. To do so, we first select the Polymod matrices, correct for reciprocity in each of them (assuming demographies of the year 2016) and normalize. Then we build an average Polymod matrix as:

$$M_{i,j}^{(Polymod)} = \frac{\sum_c M_{i,j}^{(c)} n_i^{(c)}}{\sum_c n_i^{(c)}} \tag{31}$$

where the superindex c indicates the specific country of the Polymod study, and $n_i^{(c)}$ is the number of participants of age i in country c. Then, we extract $\Gamma_{i,j}^{(Polymod)}$ assuming as demography the sum over all Polymod countries. Using this matrix, we obtain projections for the other non-Polymod countries (using either M2 or M3, which are equivalent for the sake of these analyses).

Once we obtained our own trans-national projections of the Polymod data, we can compare them against the matrices proposed in[16] as well as against the empirical data reported in the different surveys conducted in the eight countries listed in table B. Importantly, the projections based on our method, which are based on the age-distributions of the countries analyzed alone, correlate very strongly with the projections proposed in[16] (r>0.9 everywhere but in Russia), even though these precise a considerably larger amount of detailed data to be built. Even more surprisingly, we see how our projections correlate slightly better to the actual contact patterns observed in empirical studies conducted in these different countries, pointing to the fact that, among all sources of heterogeneity in social mixing, age is arguably more important than other considerations that seemingly play secondary roles. However, correlations between projections and empiric data are not always consistent, but, perhaps unsurprisingly, only high enough when Polymod data is projected into other European settings (Russia and France). This warns once again against the indiscriminate extrapolation of contact structures measured in specific geographical areas to settings belonging to other continents or characterized by profound cultural or demographic differences.

References

[1] Guillaume Béraud, Sabine Kazmercziak, Philippe Beutels, Daniel Levy-Bruhl, Xavier Lenne, Nathalie Mielcarek, Yazdan Yazdanpanah, Pierre-Yves Boëlle, Niel Hens, and Benoit Dervaux. The french connection: the first large population-based contact survey in france relevant for the spread of infectious diseases. *PloS one*, 10(7):e0133203, 2015.

[2] Yoko Ibuka, Yasushi Ohkusa, Tamie Sugawara, Gretchen B Chapman, Dan Yamin, Katherine E Atkins, Kiyosu Taniguchi, Nobuhiko Okabe, and Alison P Galvani. Social contacts, vaccination decisions and influenza in japan. *J Epidemiol Community Health*, pages jech–2015, 2015.

[3] Jonathan M Read, Justin Lessler, Steven Riley, Shuying Wang, Li Jiu Tan, Kin On Kwok, Yi Guan,

Chao Qiang Jiang, and Derek AT Cummings. Social mixing patterns in rural and urban areas of southern china. *Proceedings of the Royal Society of London B: Biological Sciences*, 281(1785):20140268, 2014.

[4] UN. Population division database. *http://esa.un.org/unpd/wpp/index.htm*, (accessed November 2016).

[5] Joël Mossong, Niel Hens, Mark Jit, Philippe Beutels, Kari Auranen, Rafael Mikolajczyk, Marco Massari, Stefania Salmaso, Gianpaolo Scalia Tomba, Jacco Wallinga, et al. Social contacts and mixing patterns relevant to the spread of infectious diseases. *PLoS medicine*, 5(3):e74, 2008.

[6] Marco Ajelli and Maria Litvinova. Estimating contact patterns relevant to the spread of infectious diseases in russia. *Journal of Theoretical Biology*, 419:1–7, 2017.

[7] Kathy Leung, Mark Jit, Eric HY Lau, and Joseph T Wu. Social contact patterns relevant to the spread of respiratory infectious diseases in hong kong. *Scientific reports*, 7(1):7974, 2017.

[8] Moses Chapa Kiti, Timothy Muiruri Kinyanjui, Dorothy Chelagat Koech, Patrick Kiio Munywoki, Graham Francis Medley, and David James Nokes. Quantifying age-related rates of social contact using diaries in a rural coastal population of kenya. *PloS one*, 9(8):e104786, 2014.

[9] Olivier le Polain de Waroux, Sandra Cohuet, Donny Ndazima, Adam Kucharski, Aitana Juan-Giner, Stefan Flasche, Elioda Tumwesigye, Rinah Arinaitwe, Juliet Mwanga-Amumpaire, Yap Boum, et al. Characteristics of human encounters and social mixing patterns relevant to infectious diseases spread by close contact: A survey in southwest uganda. *bioRxiv*, page 121665, 2017.

[10] Alessia Melegaro, Emanuele Del Fava, Piero Poletti, Stefano Merler, Constance Nyamukapa, John Williams, Simon Gregson, and Piero Manfredi. Social contact structures and time use patterns in the manicaland province of zimbabwe. *PloS one*, 12(1):e0170459, 2017.

[11] Maria A Riolo and Pejman Rohani. Combating pertussis resurgence: One booster vaccination schedule does not fit all. *Proceedings of the National Academy of Sciences*, 112(5):E472–E477, 2015.

[12] Ana I Bento, Aaron A King, and Pejman Rohani. A simulation study on the relative role of age groups under differing pertussis transmission scenarios. *bioRxiv*, page 247007, 2018.

[13] Sergio Arregui, María José Iglesias, Sofía Samper, Dessislava Marinova, Carlos Martin, Joaquín Sanz, and Yamir Moreno. Data-driven model for the assessment of mycobacterium tuberculosis transmission in evolving demographic structures. *Proceedings of the National Academy of Sciences*, 2018.

[14] Mark EJ Newman. Mixing patterns in networks. *Physical Review E*, 67(2):026126, 2003.

[15] Giancarlo De Luca, Kim Van Kerckhove, Pietro Coletti, Chiara Poletto, Nathalie Bossuyt, Niel Hens, and Vittoria Colizza. The impact of regular school closure on seasonal influenza epidemics: a data-driven spatial transmission model for belgium. *BMC infectious diseases*, 18(1):29, 2018.

[16] Kiesha Prem, Alex R Cook, and Mark Jit. Projecting social contact matrices in 152 countries using contact surveys and demographic data. *PLoS computational biology*, 13(9):e1005697, 2017.
